# Surface functionalization strategies for polydiacetylene-based colorimetric sensors

**DOI:** 10.1038/s42004-026-01913-y

**Published:** 2026-01-30

**Authors:** Brainy Happy Ana Tasiman, Rizky Aflaha, Wiyogo Prio Wicaksono, Ganjar Fadillah, Yuliyan Dwi Prabowo, Joan Daniel Prades, Erwin Peiner, Kuwat Triyana, Hutomo Suryo Wasisto

**Affiliations:** 1 Department of Physics, Faculty of Mathematics and Natural Sciences, Universitas Gadjah Mada, Yogyakarta, Indonesia; 2 Department of Chemistry, Faculty of Mathematics and Natural Sciences, Universitas Islam Indonesia, Yogyakarta, Indonesia; 3 Institute of Semiconductor Technology (IHT) and Laboratory for Emerging Nanometrology (LENA), Technische Universität Braunschweig, Braunschweig, Germany; 4 PT Sciencemind Education Lab, Yogyakarta, Indonesia; 5 PT Biostark Analitika Inovasi, Bandung, Indonesia

**Keywords:** Synthesis and processing, Sensors and biosensors

## Abstract

Polydiacetylene (PDA)-based colorimetric sensors offer a promising platform for rapid and visual detection, through a chromatic transition from blue to red. However, their broader applications are hindered by challenges in sensitivity, selectivity, and stability. This review comprehensively overviews functionalization strategies to overcome existing limitations, including chemical modification with reactive groups, conjugation with specific ligands or receptors, and integration with nanomaterials. Alternative approaches are also discussed. The interplay between base materials, deposition methods, and functionalization efficiencies is emphasized. Furthermore, this review addresses remaining challenges, proposes feasible solutions, and offers insights into future strategic directions for creating more advanced PDA-based colorimetric sensors.

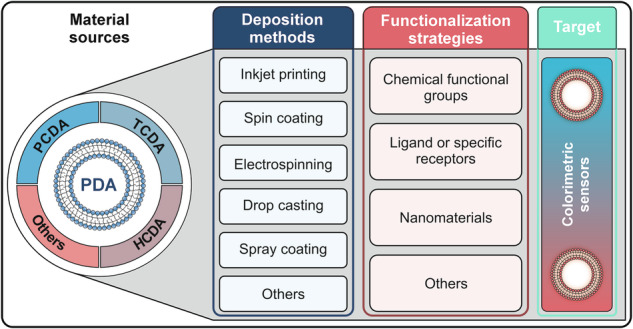

## Abstract (web summary)

Polydiacetylene (PDA)-based colorimetric sensors have potential for rapid visual detection. However, their broader application remains limited by issues of sensitivity, selectivity, and stability. Here, the authors review functionalization strategies to overcome existing limitations, including chemical modification with reactive groups, conjugation with specific ligands or receptors, and integration with nanomaterials.

## Introduction

Polydiacetylene (PDA)-based colorimetric sensors represent a highly promising technology with applications across diverse fields, including gas detection^1–6^, biomolecular analysis^7–10^, environmental pollutant monitoring^11–13^, and food safety^14–18^. These sensors operate based on visually observable color changes triggered by specific interactions or stimuli, such as temperature^19–21^, pH^6^, or the presence of target molecules^22–26^. PDA-based sensors offer a rapid and straightforward detection process that does not require complex instrumentation^27–30^. Despite these advantages, PDA-based sensors often suffer from limited sensitivity and selectivity^31,32^. They tend to exhibit cross-reactivity with structurally similar compounds and struggle to detect analytes at trace levels, significantly hindering their practical applications^33^.

A critical determinant of sensor performance lies in the employed functionalization strategy. Modifying the PDA structure through chemical or molecular techniques has been shown to enhance sensitivity, specificity, and environmental stability^32,34^. Functionalization approaches include chemical grafting with carboxylic acid groups^35^, amines^36^, or thiols^37,38^, to enhance molecular recognition, as well as the immobilization of selective receptors such as aptamers^7^ and peptides^39^. Additionally, integrating PDA with nanomaterials for signal amplification, such as gold nanoparticles^40^, zinc oxide^41^, or graphene oxide^42^, has demonstrated significant potential for improving detection performance. Recent developments have introduced more sophisticated functionalization strategies, significantly enhancing the capabilities of PDA-based colorimetric sensors. Notable examples include PDA-aptamer hybrids for detecting pathogens and disease biomarkers^7,43,44^, as well as amine-functionalized PDA for hazardous gas detection^36^. In particular, PDA systems with amine groups have undergone further development in recent years, such as the creation of PDA-functionalized polyamine vesicle designs for carbon dioxide (CO_2_) detection that provide better sensitivity and stability^36^. More recently, integrated all-in-one detection platforms have been reported, such as microneedles (MNs) complexed with dopamine-functionalized PDA and amino-functionalized PDA to detect physiological targets with higher sensitivity^45^. However, many prior studies focused narrowly on material composition and its specific application, often overlooking the critical interplay among material sources, fabrication techniques, and functionalization strategies. In reality, these parameters are deeply interconnected and collectively influence overall sensor performance.

Compared to other materials used in colorimetric sensors, such as gold nanoparticles (AuNPs)^46–50^ and organic dyes^51–53^, PDA offers unique advantages due to its conjugated backbone when responding to targets^54–56^. AuNPs are indeed known for their high stability and good biocompatibility. However, their use is often hindered by complex synthesis and functionalization processes, as well as challenges related to sensitivity, specificity, and reproducibility that still require attention^57–59^. On the other hand, organic dyes are susceptible to photobleaching and environmental interference, and have limited sensitivity in complex matrices^53,60,61^. Unlike AuNPs and organic dyes, PDA combines ease of fabrication, strong stability, and modifiable chromatic properties, enabling its widespread use in various sensor applications^5,62–69^. The sharp and significant color transition when interacting with a target enables real-time detection in the field without requiring advanced equipment^28,30,60,70^. Additionally, PDA’s compatibility with various functionalization strategies enables the design of customizable sensors to meet specific detection needs more practically^16,71,72^.

Accordingly, we provide a comprehensive overview of functionalization strategies in PDA-based colorimetric sensors, emphasizing the integration of material synthesis, fabrication methodologies, and functionalization pathways. It examines the strengths and limitations of various strategies within specific applications, compares different fabrication techniques, and explores the structure-performance relationships in PDA systems. Furthermore, it highlights recent innovations, such as the integration of PDA sensors with smartphones for portable sensing solutions. Given the growing demand for rapid, portable, and cost-effective sensing technologies, particularly in medical diagnostics, environmental monitoring, and food safety, this review aims to guide future research directions and foster meaningful innovations in PDA-based colorimetric sensors.

## Polydiacetylene-based colorimetric sensor arrays

### Working principle

**Fig. 1:**
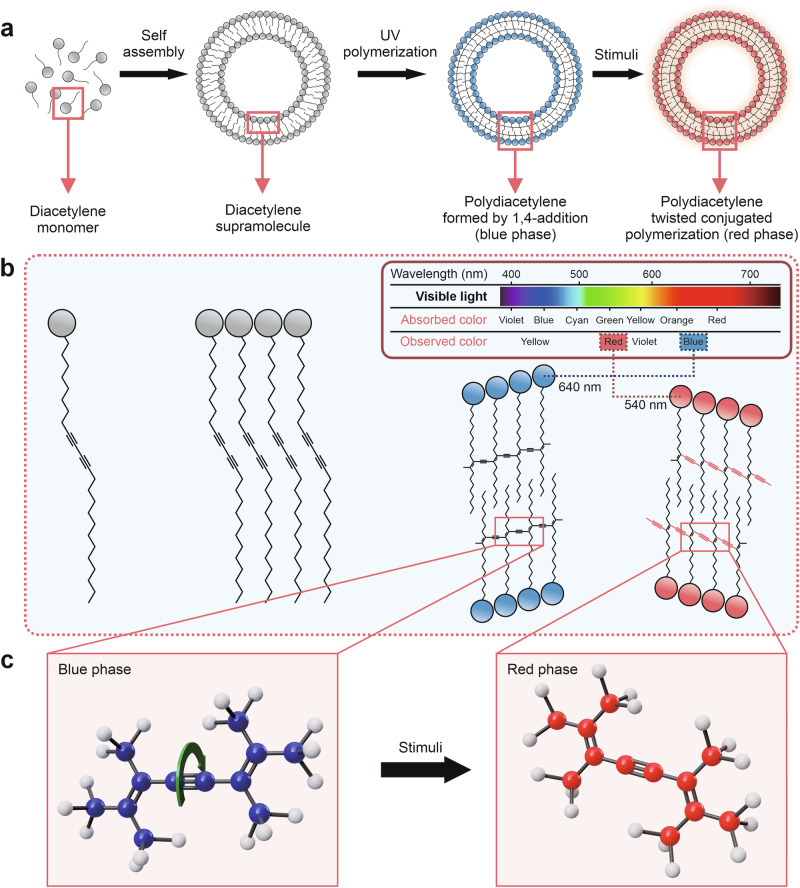
Working principle of polydiacetylene (PDA)-based colorimetric sensors. **a** Self-assembly of amphiphatic diacetylene monomers into highly ordered diacetylene supramolecules, followed by UV polymerization (254 nm) to form blue-phase polydiacetylene (PDA) with a conjugated backbone, which undergoes a color change to red-phase polydiacetylene when exposed to environmental stimuli. **b** Molecular structure of PDA chain, showing the transformation from regular conjugated backbone (blue phase) to a twisted conformation (red phase). Adapted with permission from ref. ^76^, copyright (Elsevier B.V., 2021). **c** The rotation representation on the PDA backbone causes conversion from the blue to red phases. Adapted with permission from ref. ^77^, copyright (American Chemical Society, 2018).

PDA-based colorimetric sensors operate on the unique optical properties of PDA polymers. The polymerization of diacetylene (DA) monomers into PDA generally occurs through a self-assembly process followed by UV-induced topochemical polymerization, which proceeds without the need for chemical initiators. This method enables synthesis with high purity and minimal byproducts^42,73^. Fundamentally, the performance of PDA-based colorimetric sensors is closely associated with the molecular design of the DA monomer. The amphiphilic nature of the monomer is a critical determinant of its ability to self-assemble into regular supramolecular structures, a prerequisite for topochemical polymerization. Upon UV irradiation (254 nm), 1,4-addition polymerization produces a polymer backbone with a conjugated structure characterized by alternating double (C = C, *ene*) and triple (C ≡ C, *yne*) bonds along the chain, as illustrated in Fig. 1a^63,70,71,74^. This conjugated structure creates an extensively delocalized π-electron system along the polymer chain, resulting in the characteristic blue color of PDA, with an absorption peak at ~640 nm, located within the red region of the electromagnetic spectrum but perceived visually as blue. The stability of this blue phase is entirely dependent on the molecular order established during self-assembly. Thus, any disruption in molecular packing directly affects its optical properties^63,74^.

Within the conjugated system, the primary detection mechanism involves a color transition from blue to red, which is generally attributed to electronic changes arising from distortion of the polymer backbone conformation^23–25^, In the blue phase, the planar polymer chain allows extensive π-electron delocalization, resulting in a relatively narrow highest occupied molecular orbital-lowest unoccupied molecular orbital (HOMO-LUMO) band gap. When the sensor interacts with an analyte or target via electrostatic forces, dipole-dipole interactions, or steric hindrance, the initially planar backbone becomes distorted into a non-planar configuration. This distortion disrupts π-electron delocalization, widens the band gap, and shifts the absorption spectrum toward higher energies (~540 nm), manifesting visually as a red color, as illustrated in Fig. 1b, c. This band-gap expansion is spectroscopically characterized by a hypsochromic shift (blue shift) of the absorption peak from ~640 to ~540 nm^74^. Because the material now absorbs higher-energy blue-green light, the visible color changes to red.

In addition to its characteristic color transition, PDA also exhibits fluorescence emission when structural disruption occurs. The blue phase is generally non-fluorescent, whereas the red phase shows red fluorescence. This behavior is associated with an energy shift in the lowest excited state as the material transitions from the blue to the red phase. Consequently, PDA-based systems can generate dual optical responses, both colorimetric changes and fluorescence emission, making them attractive and multifunctional candidates for sensor platforms^63,67,75^. The chromatic transition of PDA can be observed directly with the naked eye or quantified using analytical techniques such as image analysis, UV–Vis spectrophotometry, or fluorescence spectroscopy^71–77^.

**Fig. 2:**
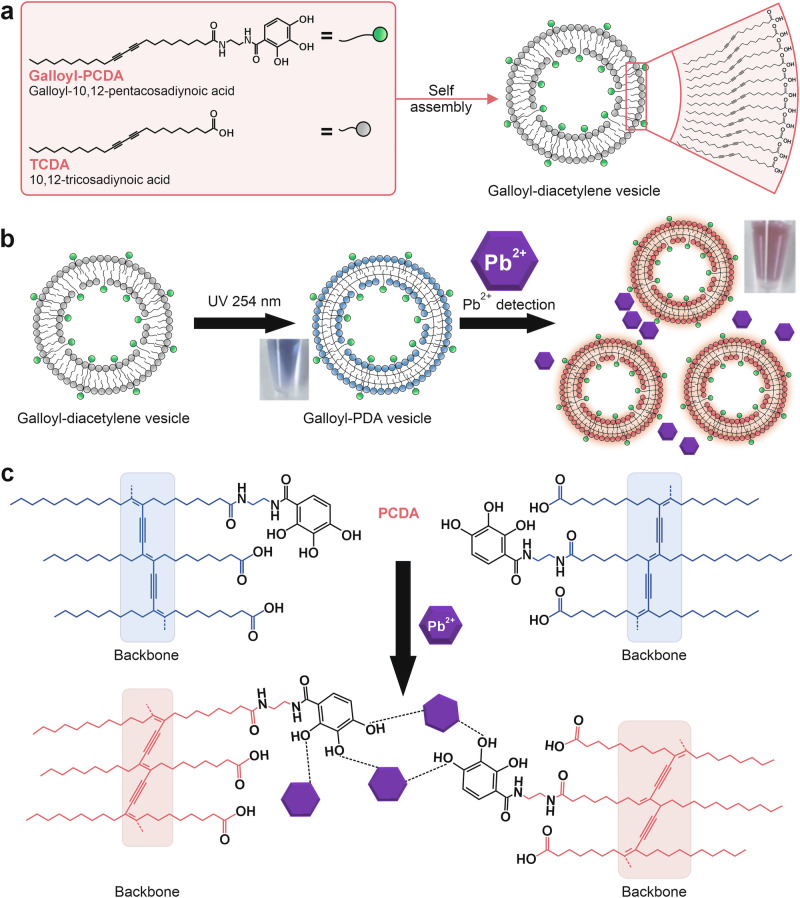
Polydiacetylene (PDA)-based colorimetric sensor functionalized with galloyl groups for Pb²⁺ ion detection. **a** Structure of diacetylene vesicles functionalized with galloyl groups, which assemble to form galloyl-PDA vesicles. **b** Dual-signal detection of Pb^2+^ by blue galloyl-PDA vesicles polymerized upon 254 nm UV irradiation. **c** Detection mechanism of Pb^2+^ by galloyl-PDA vesicles, where Pb^2+^ interacts with galloyl-PDA vesicles, triggering conformational distortion of the PDA backbone. Adapted with permission from ref. ^82^, copyright (Elsevier B.V., 2022).

PDA-based colorimetric sensors are typically fabricated by depositing PDA derivatives onto various substrates. These derivatives are functionalized with specific chemical headgroups that impart selective affinity toward target analytes. The structural diversity of these headgroups allows the PDA matrix to generate distinct optical responses upon interaction with different analytes, including metal ions, gases, and biomolecules. The interaction between analytes and the PDA’s functional groups induces the characteristic blue-to-red color change, creating a unique colorimetric fingerprint for each target compound^78^. These color transition mechanisms include specific interactions such as hydrogen bonding, electrostatic attraction, or covalent bonding between the analyte and the sensor’s functional moieties^67,79^. PDA’s chromatic adaptability stems from the modifiable nature of the DA monomer’s side chains. By tailoring the hydrophilic headgroup, researchers can enhance the sensor’s selectivity and sensitivity toward particular analytes^80,81^. Incorporating functional detection probes directly into the DA headgroup has been shown to enhance the intensity and specificity of the PDA’s chromatic response^77^. These interaction-induced changes produce analyte-specific response patterns, which can be analyzed through qualitative observation and quantitative data processing. An illustrative example is the development of a PDA-based sensor functionalized with galloyl groups to detect Pb²⁺ ions. As illustrated in Fig. 2a–c, the interaction of Pb^2+^ with galloyl-PDA induced a conformational rearrangement of the conjugated backbone, resulting in an electronic transition that produced a distinct colorimetric change from blue to red^82^.

### Base materials

The fabrication of PDA-based colorimetric sensors necessitates a careful selection of materials to ensure optimal sensitivity, selectivity, stability, and production efficiency. The primary components of these systems include a DA monomer as the PDA precursor, a substrate for PDA immobilization, and functionalization agents that impart target-specific recognition capabilities. The diacetylene monomer plays a central role in forming the PDA’s conjugated polymer backbone, which exhibits characteristic chromatic transitions. These monomers possess a general structure with two consecutive triple bonds (R_1_–C ≡ C–C ≡ C–R_2_), allowing 1,4-topochemical polymerization into long, rigid, and highly conjugated chains upon exposure to UV light^64,74,82–84^. Furthermore, the headgroup of the DA monomer can be chemically modified to facilitate functionalization and enhance specificity toward different analytes.

**Fig. 3:**
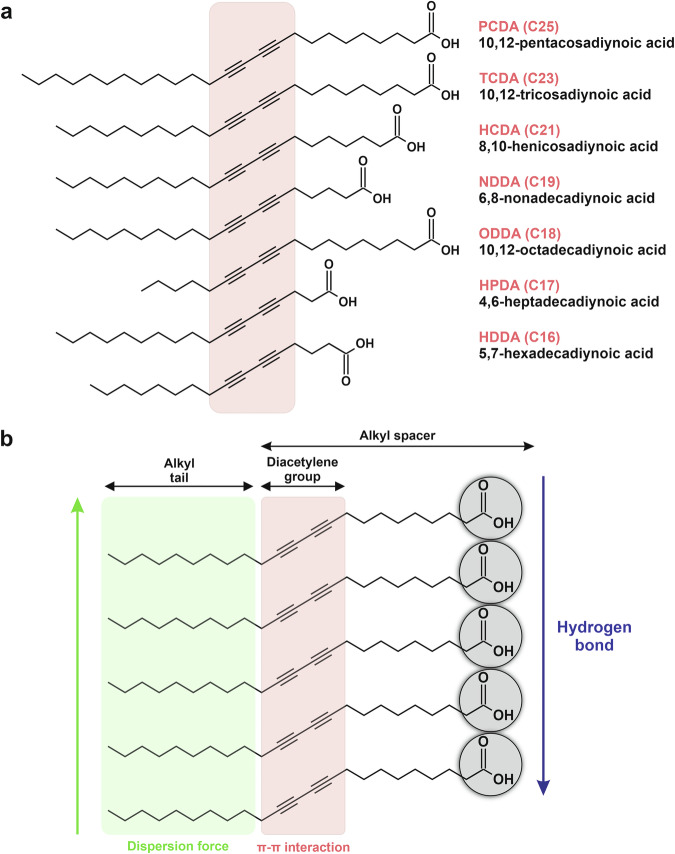
Chemical structure and intermolecular interactions of diacetylene monomers (DA) as the base material for PDA-based colorimetric sensors. **a** Various diacetylene (DA) monomers with different alkyl chain lengths (C16 – C25) serve as precursors for PDA-based colorimetric sensors. **b** Key molecular interactions within PDA assemblies include hydrogen bonds, dispersion forces, and π-π interactions. Adapted with permission from ref. ^76^, copyright (Elsevier B.V., 2021).

One of the most widely used PDA monomers (see Fig. 3a) is 10,12-pentacosadiynoic acid (PCDA), which features a 25-carbon alkyl chain and a terminal carboxylic acid (-COOH) group. PCDA is frequently employed across different applications, including hazardous gas detection, biomolecule sensing, and food safety monitoring, due to its excellent stability, high sensitivity, ease of functionalization, and pronounced chromatic response^85,86^. However, PCDA’s relatively high cost and the need for stringent synthesis control can be considered limitations.

Another commonly used monomer is 10,12-tricosadiynoic acid (TCDA), which features a slightly shorter 23-carbon alkyl chain (C23). TCDA tends to form sensor arrays with higher molecular density and exhibits enhanced sensitivity to intermolecular interactions, making it well-suited for high-temperature applications. Nevertheless, TCDA sometimes displays lower homogeneity in self-assembly compared to PCDA^87^. In addition, N-hydroxysuccinimide ester-functionalized DA (DA-NHS), a derivative of PCDA or TCDA, introduces an active ester group that enables facile conjugation with primary amines found in biomolecules such as peptides and proteins. This monomer is highly reactive and particularly advantageous in biosensing applications due to its efficient and stable covalent linkage formation^18,83,88^. Several other DA monomers, including 8,10-heneicosadiynoic acid (HCDA), 6,8-nonadecadiynoic acid (NDDA), 10,12-octadecadiynoic acid (ODDA), 4,6-heptacadiynoic acid (HPDA), and 5,7-hexadecadiynoic acid (HDDA), offer additional versatility. As illustrated in Fig. 3a, these monomers possess shorter alkyl chains than PCDA and TCDA.

Each monomer exhibits distinct properties due to inherent differences in dispersive forces, hydrogen bonding capabilities, and π–π interactions within its molecular structures (see Fig. 3b). Typically, PDAs synthesized from longer-chain monomers exhibit greater structural stability and stimulus responsiveness due to the enhanced supramolecular order that occurs during self-assembly and polymerization. Nevertheless, the functional performance of short-chain DA monomers can be significantly improved through surface modification or nanocomposite formation strategies^76,89^. For instance, a study by Yimkaew et al. demonstrated that the integration of Zn²⁺ ions or ZnO nanoparticles with short-chain DA monomers (HPDA and HDDA) significantly enhanced sensor performance for detecting low-concentration surfactants. This enhancement was attributed to reduced dispersive interactions, allowing surfactants to more readily penetrate the PDA bilayer structure easily^41^. Therefore, the selection of DA monomers must be tailored to meet the demands of specific sensing applications^90^.

In addition to the alkyl-chain length, the chemical properties of the DA monomer head group also play a crucial role in determining sensor performance^75,81,91^. The molecular structure, intermolecular interactions (e.g., hydrogen bonding, electrostatic forces), steric effects, and charge state of the head group collectively guide the self-assembly of monomers into a highly ordered structure, an essential prerequisite for successful topochemical polymerization into the blue phase with optimal optical characteristics. Because the head groups directly participate in assembly and polymerization, their structural design significantly affects the rigidity of the conjugated backbone, the stability of the blue phase, and the ease of triggering the blue-to-red color transition^75,92–97^. Furthermore, the head group acts as a recognition site capable of interacting specifically with target analytes through non-covalent interactions (e.g., hydrogen bonding, π–π stacking, van der Waals forces) or direct chemical reactions^63^. These interactions perturb the PDA backbone, thereby inducing the characteristic chromatic transition.

Consequently, the rational design of the head group is essential for regulating interactions between PDA and the target analyte, directly determining the sensitivity and selectivity of the resulting colorimetric response^6,55,63,98,99^. For instance, various DA monomers have been synthesized with functional groups such as amines, amides, imidazoles, and triazoles to enable PDA-based acid sensing^6,55,98,99^. Saymung et al. reported that converting the carboxylate group (–COOH) on PDA into its sodium salt form (–COO⁻Na⁺) produced a sensor responsive to hydrochloric acid (HCl)^55^. Upon acid exposure, protonation of –COO⁻Na⁺ to –COOH alters local interactions, inducing conformational distortions in both the conjugated backbone and the side chains and resulting in a pronounced color change. Additionally, the presence of –COO⁻Na⁺ enhances thermal stability due to strong electrostatic interactions^55^.

### Deposition methods

The effectiveness of PDA-based colorimetric sensors is strongly influenced by the fabrication or deposition technique used to apply the PDA layer, as this determines its uniformity, stability, and functional responsiveness. Various fabrication methods have been developed (see Table 1), each offering distinct advantages depending on the sensor’s intended applications and performance requirements. Commonly employed techniques include spin coating, inkjet printing, screen printing, electrospinning, drop casting, and spray coating^62^. In general, these methods involve the formation of diacetylene supramolecular assemblies followed by UV-induced polymerization to form the blue-phase PDA. The choice of deposition method must align with the sensor’s operational objectives, considering factors such as substrate compatibility, spatial resolution, ease of processing, and cost efficiency.

**Table 1:** Various techniques to fabricate polydiacetylene (PDA)

Method	PDA materials	Benefits	Limitations	Applications	Refs.
Spin coating	• Diacetylene monomers (e.g., PCDA and TCDA) • Solvents (e.g., tetrahydrofuran and dicloromethane)	• Excellent PDA film homogeneity • Consistent, fast, efficient, and reproducible optical response	• Not suitable for complex patterns • Strict process control required.	Environmental monitoring	^100,101^
Inkjet printing	• Diacetylene monomers (e.g., PCDA, TCDA) • Solvents (e.g., tetrahydrofuran)	• Easy, cheap, and highly precise process • Adjustable printing patterns • Efficiently used materials	• Limited ink formulation • Easily clogged nozzles	Gas sensing	^28,29^
Electrospinning	• Diacetylene monomers (e.g., PCDA and TCDA) • Solvent (e.g., dimethylformamide)	• High specific surface area • Clearer and faster color response than film • Stable and uniform performance	• Complex process requiring special equipment • Limited types of applicable substrates	Gas sensing	^42,73,102–106^
Drop casting	• Diacetylene monomer (e.g., PCDA and TCDA) • Solvent (e.g., ethanol and chloroform)	• Simple and low-cost process • High portability	• Low film homogeneity • Low suitability for high-precision applications	Gas and vapor sensing and environmental monitoring	^112–116^
Spray coating	• Diacetylene monomer (e.g., PCDA) • Solvent (e.g., ethanol)	• Fast and low-cost process • High suitability for large or flexible substrates	• Limited thickness and distribution control	Gas sensing and environmental monitoring	^1,87^

Each PDA fabrication method has its own benefits, limitations, and applications. Typically used diacetylene monomers are 10,12-pentacosadiynoic acid (PCDA) and 10,12-triacosadiynoic acid (TCDA).

Spin coating is one of the most widely used methods for producing uniform PDA thin films on substrates such as glass, polyethylene terephthalate, or polydimethylsiloxane (PDMS). In this technique, a diacetylene monomer solution dissolved in an organic solvent is dispensed onto the substrate, which is then rotated at high speed to achieve uniform film distribution. This approach enables precise control over film thickness and surface homogeneity, both of which are crucial for achieving a consistent chromatic response. Spin-coated PDA films are particularly well-suited for gas and vapor sensing applications that require sharp and rapid color transitions. However, spin coating is less suitable for creating complex patterned structures and faces scalability challenges due to its sensitivity to process parameters^100,101^.

**Fig. 4:**
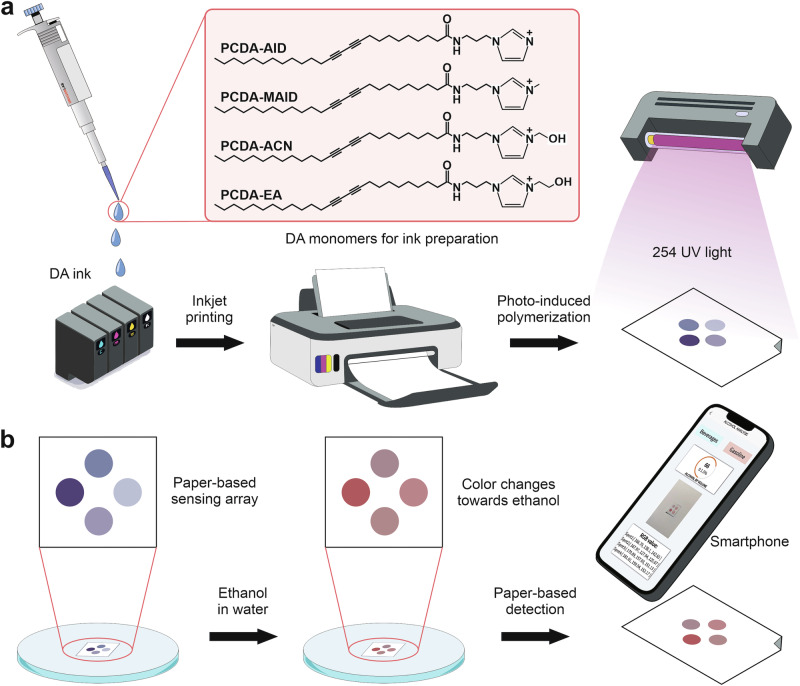
A paper-based polydiacetylene (PDA) sensor array fabricated by inkjet printing method. **a** Fabrication process of the paper-based PDA sensor arrays by light-induced polymerization under 254 nm UV light. **b** Detection of ethanol in water using the printed PDA sensor array and smartphone. Adapted with permission from ref. ^29^, copyright (Elsevier B.V., 2024).

In contrast, inkjet printing offers a highly versatile and scalable approach for fabricating PDA-based sensors, especially for flexible and disposable formats, as illustrated in Fig. 4. In this method, the DA monomer is formulated into a printable ink and deposited directly onto a substrate in defined patterns (see Fig. 4a, b). Inkjet printing enables direct, precise, and customizable patterning on a variety of substrates with minimal material waste. It is particularly advantageous for the fabrication of paper-based and flexible sensors, combining production scalability, low cost, and design flexibility. As a result, inkjet printing is often preferred for the development of portable and mass-producible PDA colorimetric sensors^28,29^.

Other deposition methods also provide unique advantages for specific applications. For example, electrospinning produces PDA nanofibers with a high specific surface area, enhancing analyte interaction and improving colorimetric response time and intensity. This method is particularly suitable for gas sensing and biomedical applications that demand high sensitivity, although it requires complex material processing and specialized equipment with high voltage supply in the range of a few tens to hundreds of kilovolts (kV)^42,73,102–111^. Drop casting is a simple and cost-effective technique, in which a PDA monomer solution is dropped directly onto a substrate and allowed to dry. Although this method offers easy implementation and portability, it often results in poor film uniformity and limited reproducibility, making it less appropriate for high-precision applications^112–116^. Spray coating offers a rapid and economical option for fabricating large-area or large-scale sensors. However, this technique offers limited control over film thickness and uniformity, which may affect reproducibility and sensor sensitivity^1,87^.

Ultimately, the selection of a suitable deposition method must be integrated with the sensor’s functionalization strategy and performance goals. Each technique requires careful optimization of material parameters, such as solvent type, viscosity, and monomer concentration, to ensure proper dispersion, rheological behavior, and substrate adhesion. These parameters directly affect the final PDA morphology and its chromatic response. Therefore, a holistic approach that integrates material synthesis, surface functionalization, and deposition is essential to optimize the overall performance of PDA-based colorimetric sensors. A comparative summary of the key characteristics of each deposition technique is provided in Table 1.

### Color readout methods

While simple visual checks may suffice for ultra-low-cost use, automating readout unlocks clear gains, i.e., objective and repeatable color assessment, accessibility for color-impaired users, automatic data logging and traceability, machine-based decision making, extraction of patterns from multiplexed arrays that are intractable to the human eye, and ultimately rigorous sensor quantification. Today’s solutions span from phones to professional optics.

At the low-cost end, smartphone imaging in light-shielded holders or boxes, combined with reference targets and algorithmic correction, delivers quantitative readout on disposable substrates^117,118^. Recent systems image full microplates with onboard or server-side analysis, enabling classification and quantification^119,120^. Open, low-cost designs further lower barriers to deployment^121^. Increasingly, pipelines incorporate machine learning for robust segmentation and color–concentration mapping under varied lighting and device conditions^122^. In this context, color calibration is pivotal, where recent works show that back-compatible color quick response (QR) codes embed hundreds of reference patches directly in the field of view, simultaneously carrying digital metadata and enabling per-image color correction that reduces inter-device error and improves reproducibility^123,124^. These “calibration-in-scene” strategies are backward-compatible with standard QR decoding, easing adoption in packaging and point-of-use assays.

For tighter metrology—calibration transfer, weak chromatic shifts, or regulatory contexts—portable spectrophotometers and spectrocolorimeters provide full-spectral capture, higher precision, and traceable performance. Comparative studies show that modern handheld spectrophotometers generally outperform red, green, and blue (RGB) camera systems for color accuracy while remaining field-deployable^125^. Portable colorimeters have also proven effective for routine, repeatable measurements in specific applications^126^. Across this spectrum, best practice couples controlled illumination (or embedded references) with automated image/signal processing to deliver accurate, operator-independent readout and seamless digitization of colorimetric data.

## Advanced surface functionalization strategies

The performance of PDA-based colorimetric sensors can be significantly improved through advanced surface functionalization strategies. Each strategy offers a distinct mechanism for modifying the PDA structure, thereby enhancing sensitivity, selectivity, and stability toward specific target analytes. Broadly, three major functionalization approaches are commonly used: (1) chemical functionalization with specific groups, which involves specific interactions such as electrostatic attraction or metal-ion coordination; (2) conjugation with ligands or specific receptors, which relies on the high affinity of selected biomolecules; and (3) nanomaterial integration, which leverages the unique properties of nanomaterials to improve signal amplification and structural stability. Beyond these three primary approaches, additional methods, such as the formulation of PDA into lipid vesicles through self-assembly and UV-induced polymerization, have also been explored to increase sensor versatility. All surface functionalization strategies will be critically examined by comparing their advantages, limitations, and potential applications in detection systems.

### Chemical functional groups

**Fig. 5:**
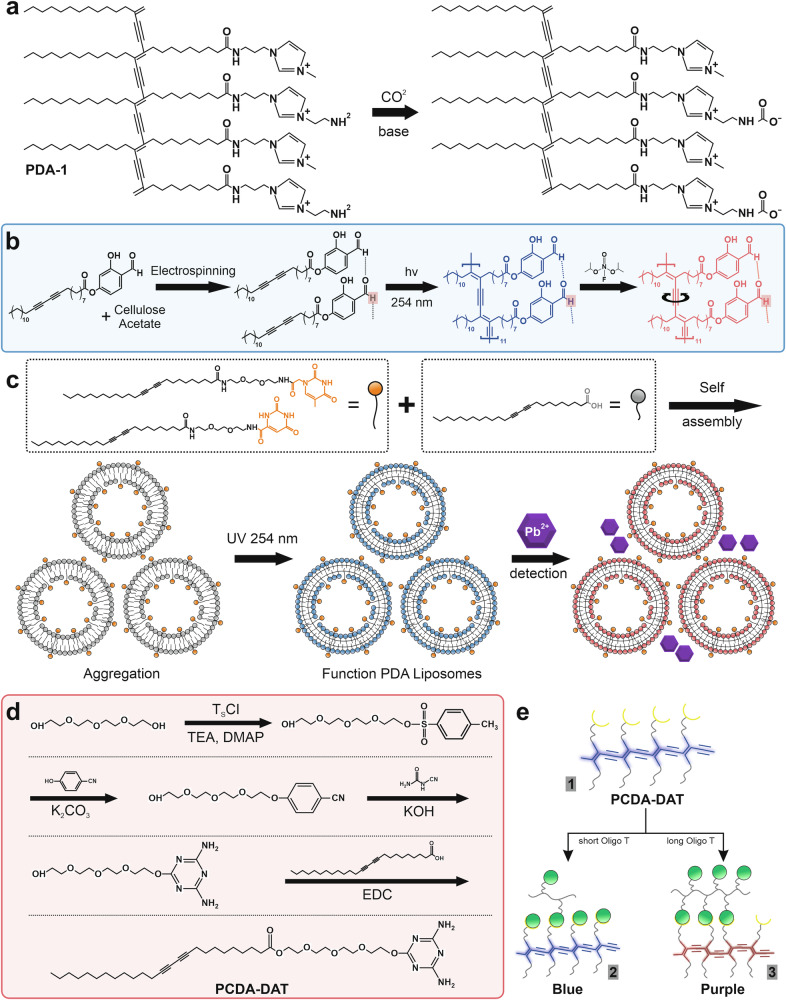
Surface functionalization of polydiacetylene (PDA)-based colorimetric sensors using chemical functional groups. **a** Functionalization with amines and imidazolium groups for CO_2_ detection, which react to form carbamate anions, triggering a blue-to-red color transition. Adapted with permission from ref. ^128^. Copyright 2013 American Chemical Society. **b** Functionalization with ester for organophosphate pesticide detection, where hydrogen and covalent bonds facilitate a color shift. Adapted from ref. ^131^, copyright (American Chemical Society, 2025). **c** Functionalization with thymine-1-acetic acid (TAA) and orotic acid (OA) groups for Pb^2+^ detection through coordination interaction with carbonyl and nitrogen groups, leading to stable complex formation and a distinct color change. Adapted from ref. ^132^, copyright (Royal Society of Chemistry, 2025). **d** Functionalization with diaminotriazine (DAT) for the selective detection of thymine groups through the formation of specific hydrogen bonds between the carbonyl (C = O) and amino (N–H) groups of thymine. **e** Colorimetric response of PDA-DAT functionalization. Adapted with permission from ref. ^135^, copyright (Elsevier B.V., 2023).

Chemical modification of PDA-based colorimetric sensor materials plays a crucial role in enhancing their sensitivity and selectivity to specific target analytes or compounds. This improvement is achieved through the addition of specific functional groups that can form selective interactions, such as hydrogen bonds, electrostatic attractions, or coordination with metal ions^71,75,127^. Several chemical modifications with specific functional groups are illustrated in Fig. 5a–e. One example of successful chemical modification strategies is a study conducted by Kim et al.^36^, in which they modified a PDA vesicle-based colorimetric sensor with polyamine for CO_2_ detection. The diacetylene monomer of 10,12-tricosadiynoic acid (TRCDA) was activated using N-hydroxysuccinimide (NHS), then reacted with diethylenetriamine (DETA) through an amide bond. The sensor exhibited a blue to red color transition upon reacting with CO_2_, attributed to the formation of carbamate and H^+^ protons from the reaction of CO_2_ and the amino group of DETA, which then lowers the local pH^36,128^. Decreasing the pH causes the protonation of amino groups, which disrupts the hydrogen bonds between PDA chains, resulting in distortion of the conjugated structure and triggering a shift in absorbance from 640 nm (blue) to 550 nm (red)^16^. The resulting colorimetric response percentage (CR%) reached (34.39 ± 1.46)%, which was observed within 3 min after exposure to CO_2_. TRCDA-DETA exhibited 168 h of stability under storage conditions^36^. In a related approach, the development of PDA sensors for CO₂ detection can be achieved through functionalization with amine and imidazolium groups, as illustrated in Fig. 5a. Amines react with CO₂ under basic conditions to form carbamate anions, which neutralize part of the polymer’s positive charge, thereby triggering a phase transition that causes a color change^128^.

Another modification strategy was demonstrated by Lee et al., who functionalized PDA with oxime (OX) groups to detect organophosphate nerve agents^129^. OX was selected due to its strong nucleophilic character and high reactivity toward electrophilic species, such as organophosphate (OP)^130^. In their study, OX exhibits a high affinity for OP, so that OX reacts with OP, forming an OX-OP complex. The OX-OP complex creates steric stress on the PDA surface, inducing a conformational change in PDA, which results in a rapid and sensitive blue-to-red color shift. Their developed sensor exhibited high selectivity for diethylchlorophosphate (DCP) and diisopropylfluorophosphate (DFP), with a color change within 10 s, while remaining unreactive to strong acids, such as HCl or H_3_PO_4_. These findings highlight the potential of PDA sensors for detecting hazardous compounds^129^. Efforts to enhance detection capabilities have also been extended by incorporating PDA into solid matrices, as conducted by Alam et al.^131^. They modified PDA with aldehyde groups through esterification with 2,4-dihydroxybenzaldehyde and subsequently integrated the modified PDA into the cellulose matrix. This functionalization enables the formation of hydrogen and covalent bonds with OP compounds, leading to the distortion of the PDA conjugated chain and facilitating a color change from blue to red (see Fig. 5b)^131^. This sensor exhibits high selectivity towards OP compounds without reacting to non-OP compounds^103^.

In addition to aldehyde modification, metal ion-based strategies have also been explored, as demonstrated by Kingchok et al., who modified PDA with sodium ions (Na^+^) for real-time detection of HCl^5^. The used PDA monomer was 5,7-hexadecadynoic acid (HDDA). HDDA reacted with NaOH to convert the -COOH group on PDA to -COO-Na^+^. The interaction between Na^+^ and the carboxylate group will form organized nanoribbons through the sol-gel process. During HCl detection, HCl will protonate the -COO-Na^+^ group to -COOH and disrupt the PDA structure^5^. This process disrupts the PDA conjugation and produces a color change from red (520 nm) to orange (475 nm) within 60 s. The sensor also has high selectivity for HCl, with no response to N_2_, O_2_, CO_2_, or NH_3_, and good color stability for up to 3 weeks under storage conditions^5^. In further development, PDA-based sensors are also functionalized to detect hazardous compounds, such as formaldehyde and cyanide. Siribunbandal et al. successfully enhanced the sensitivity and selectivity to formaldehyde by combining thiol (-SH) group functionalization with the addition of ZnO particles^37^. The thiol (-SH) group increases sensitivity through covalent or electrostatic bonds, while ZnO acts as a provider of active sites (Zn^2+^ ions) that will interact with the carboxylate and thiol (-SH) groups on the PDA. The reaction that occurs causes a color change from blue to red in the concentration range of 200–900 ppm^37^. On the other hand, Lee et al. used a modification with benzylidene molonitrile (BMN) to detect cyanide through nucleophilic addition^27^. The reaction distorted the π-conjugated PDA backbone and a color change specific to cyanide, with a detection limit of 0.55 μM^27^.

PDA functionalization strategies have also been effectively applied to the detection of heavy metal ions, extending their use beyond gases and small molecules. Chen et al. reported the modification of PDA using thymine-1-acetic acid (TAA) and orotic acid (OA) to increase the selectivity towards Pb^2+^ as illustrated in Fig. 5c^132^. TAA and OA will bind Pb^2+^ through interactions with carbonyl and nitrogen groups, and form a stable complex. This reaction results in a color change from blue to red, with detection limits of 38 nM and 25 nM for PDA functionalized with TAA and OA, respectively^132^. In addition to using TAA and OA, increasing selectivity and sensitivity to Pb^2+^ can also be done using phenylboronic acid (pBA). The boronic acid (B-OH) and carboxylate (COOH) groups form a stable complex, causing a color change^133^. Meanwhile, Kaewtong et al. conducted chemical functionalization for the detection of Au^3+^ using rhodamine B^134^. In their study, rhodamine B was first functionalized with ethylenediamine to enable specific binding to the Au^3+^ metal. Au^3+^ will bind to the carbonyl and nitrogen groups in rhodamine B, which causes the opening of the spirolactam ring. This triggers the color change from pink to dark red. This sensor shows high selectivity towards Au^3+^ compared to Cu^2+^, Cr^3+^, Al^3+^, and Hg^2+^ metal ions, with a sensor detection limit of 10^-7 ^M^134^. Dual functionalization was also applied to enhance the detection capability of calcium ions (Ca^2+^), as reported by Oh et al.^71^. In their study, PDA was modified with phosphate and carboxylate groups to create a selective microenvironment for Ca^2+^, resulting in a color change from blue to purple with a detection limit of 0.97 μM.

PDA-based colorimetric sensor applications have also been developed for the biosensor field, especially in biomolecule detection. Jung et al. functionalized PDA with 9-aminoacridine (9AA) intercalator to detect double-stranded DNA (dsDNA) from PCR amplicons^86^. This modification enables the intercalation of 9AA into dsDNA, causing distortion in the PDA conjugate structure and resulting in a color change from blue to red, with a detection limit of 20 nM. In clinical applications, the sensor successfully detected BRCA1 gene amplicons from breast cancer patient samples with a fast detection time (1 h), without the need for extensive DNA purification. This sensor has great potential for point-of-care testing applications without the need for complex instrumentation^86^. Similarly, Jannah et al. successfully developed a PDA sensor functionalized with diaminotriazine (DAT) for the selective detection of thymine and oligothymidine^135^. Diacetylene monomer (10,12 pentacosadiynoic acid or PCDA) was modified with a DAT group having two amino groups (-NH_2_) that allow the formation of specific hydrogen bonds with the carbonyl (C = O) and amino (N-H) groups of thymine. This interaction triggers a conformational change in the conjugated chain on PDA, resulting in an electronic shift, as well as a change in absorbance from 642 to 542 nm. This shift is accompanied by a macroscopic color change from blue to purple, as shown in Fig. 5d, e, which provides a clear visual indication of the presence of thymine. The PDA-DAT sensor exhibits high selectivity, responding only to thymine and not to other bases, such as adenine, guanine, and cytosine, or interfering molecules, including urea and glucose. Sensitivity test showed that the limit of detection (LOD) values were 12.6 nM and 14.6 nM for thymine and oligothymidine, respectively. Fourier-transform infrared (FTIR) spectroscopy validation showed a shift in the typical peaks of NH (3445 cm^−1^ and 3350 cm^−1^) and CN triazine (1674 cm^−1^), indicating the formation of hydrogen bonds between DAT and thymine. This confirms that chemical modification through the addition of specific functional groups can significantly improve the performance of PDA-based colorimetric sensors^135^.

Beyond enhancing sensitivity and selectivity, chemical modification is also used to improve the thermal stability and reversibility of PDA sensors. PDA is known as a thermochromic material that changes color when exposed to heat. Still, most PDAs undergo irreversible color changes due to their low melting point (~63 °C) and instability at high temperatures. Modification of the hydrophilic head of PDA with urea can form intermolecular hydrogen bonds, thereby improving its thermal stability and reversibility. For example, Mapazi et al. successfully developed a PDA based on N-acylurea that remains reversible up to 150 °C, with high thermal stability up to 315 °C^19^. This stability is achieved through the formation of intermolecular hydrogen bonds that strengthen the crystal structure of PDA. Meanwhile, Han et al. conducted an in-depth study on the effect of organic functional groups and alkyl chain length on the mechanism of PDA color change^136^. This study reported that polar groups, such as -NH_2_ and -COOH, enhance intermolecular interactions (hydrogen bonds and dipole-dipole) with water, resulting in color changes at lower temperatures. In contrast, non-polar groups cause changes at high temperatures (80 °C). This study significantly contributes to the advancement of innovative materials with controlled responses^136^. The use of PDA as a low-temperature indicator is also noteworthy, as demonstrated by Goyal et al., who developed a PDA-based thermochromic indicator that functions at low temperatures (15 °C) with irreversible properties for monitoring frozen products (food and medicine)^20^. In their study, the color stability of the sensor was improved with a polyvinyl alcohol (PVA) matrix. PCDA monomers were modified through a Steglich esterification reaction with phenol (Ph). The results showed that PDA modified with phenol experienced an irreversible chromatic transition from blue to orange at a temperature of 15 °C. This is related to the absence of hydrogen bonds between ester groups (-COO-Ph). Meanwhile, in pure PDA, a chromatic transition from blue to red occurs above a temperature of 45 °C. This is due to the presence of hydrogen bonds between -COOH groups. That study is crucial for the development of more sensitive and specific PDA-based temperature sensors, particularly for packaging frozen food and pharmaceutical products^20^.

Chemical functionalization is a versatile and widely used strategy for PDA-based sensors. Its main advantage lies in the direct modification of PDA with specific interactions (e.g., electrostatic attraction or metal-ion coordination), enabling targeted interactions with analytes such as metal ions, gases, and small molecules^71,75,127^. This approach generally provides sensor designs with good batch-to-batch reproducibility and is relatively cost-effective, particularly when simple functional groups (e.g., carboxylates and Na^+^) are employed^55,71^. However, the synthesis of functionalized compounds can be complex, and their selectivity may be limited by cross-reactivity with structurally similar interferents. In addition, some sensors exhibit irreversible responses^29^. Overall, this strategy is well-suited for the development of low-cost portable sensors, environmental monitoring tools, and food-safety applications. Its adaptable chemical modification capabilities allow the creation of sensors that are not only sensitive and selective but also economical and user-friendly, making them suitable for rapid detection in industrial, biomedical, and environmental monitoring.

### Ligand or specific receptors

**Fig. 6:**
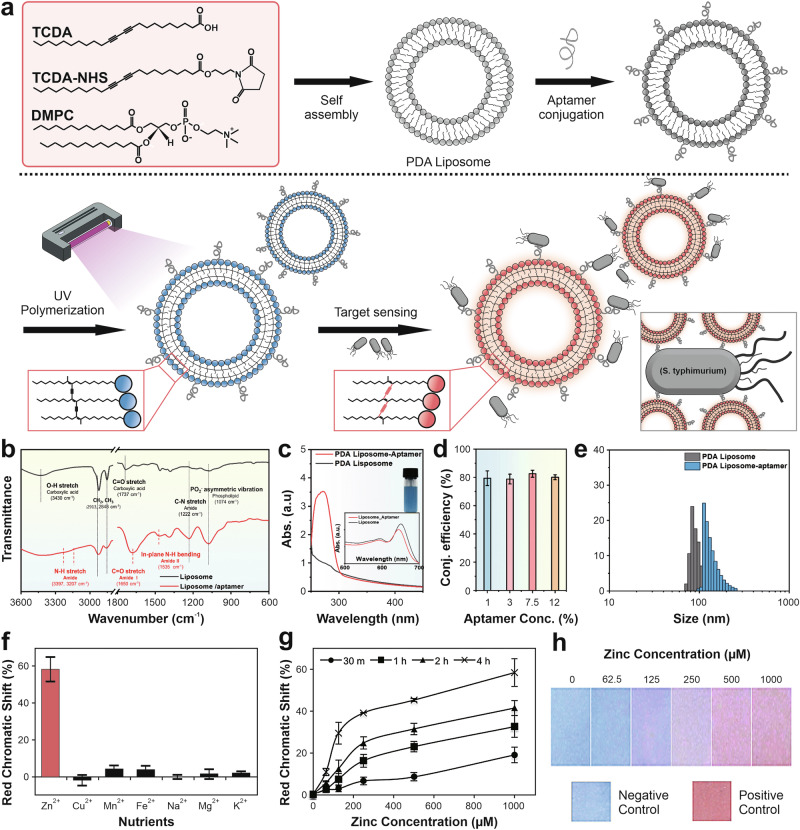
Surface functionalization of polydiacetylene (PDA)-based colorimetric sensors using specific (bio)receptors. **a** Functionalization protocol using an aptamer and corresponding sensing mechanism. PDA monomers with a molar ratio composition of TCDA, TCDA-NHS, and dimyristoylphosphatidylcholine (DMPC) of 9:1:6 were self-assembled to form PDA liposomes, followed by aptamer conjugation via a carbodiimide reaction and UV-assisted polymerization, resulting in an initial blue-colored PDA sensor. When the PDA liposome-aptamer binds specifically to the target bacteria, the binding process distorts the conjugated backbone of PDA, resulting in a visible color change from blue to red. Adapted from ref. ^7^, copyright (Royal Society of Chemistry, 2024). **b** FTIR spectra of PDA-(bio) receptor conjugations confirming successful aptamer conjugation on PDA through the appearance of characteristic amide (C = O and N–H) stretching peaks, indicating covalent-bond formation. **c** UV–Vis absorption spectra of PDA before and after aptamer attachment, illustrating the aptamer conjugation onto the surface of liposomes. **d** Conjugation efficiency of a PDA-(bio) receptor at different aptamer concentrations (1–12%), demonstrating an optimal attachment density for achieving maximum sensing response. **e** Particle-size-distribution analysis of a PDA-(bio) receptor before and after aptamer modification, demonstrating stable nanoscale structures. Reproduced from ref. ^7^, copyright (Royal Society of Chemistry, 2024). **f** Analytical performance of a PDA liposome-aptamer sensor for Zn^2+^ ion detection. The selectivity study of the PDA liposome-aptamer towards various cations showed a preferred response to Zn^2+^. **g** Red chromatic shift of PDA liposome-aptamer in response to increased Zn^2+^ concentration at different incubation times. **h** Representative color change from blue to red in PDA liposome-aptamer with increasing Zn^2+^ concentration from 0 to 1000 µM, compared to negative and positive controls. Reproduced from ref. ^139^, copyright (Elsevier B.V., 2016).

One potential approach is modification with specific ligands or receptors that can be integrated into the surface of the PDA. This strategy complements chemical modification by providing selective groups for target molecules, thereby improving sensor performance in biosensing and environmental monitoring applications. A PDA can be functionalized with specific (bio)receptors, including aptamers, oligonucleotides, antibodies, amino acids, peptides, lipids, ligands, or enzymes^7,77^. These (bio)receptors could recognize a specific analyte target on the surface of PDA sensing devices, enhancing the analytical performance of the PDA-based colorimetric sensors. Due to their high affinity and specificity, aptamers have been extensively employed in numerous sensing applications. Fundamentally, an aptamer is a bioaffinity ligand, either a peptide or an oligonucleotide aptamer, that can specifically bind to and recognize the target analyte. Protein aptamers are typically prepared using the traditional yeast hybridization system. Meanwhile, an oligonucleotide aptamer is a chemically engineered short single-stranded deoxyribonucleic acid (DNA) or ribonucleic acid (RNA) sequence (20–100 nucleotides long) synthesized through systematic ligand evolution by exponential enrichment (SELEX) techniques^137,138^. Commonly, most aptamer terminology refers to the oligonucleotide, also known as a chemical antibody, which possesses several advantages compared to traditional antibodies, such as higher binding affinity and stability, cost and time effectiveness, facile production protocol, and easy customization^138^. Lee and coworkers developed a PDA-based colorimetric sensor to detect the pathogenic bacterium Salmonella in food by functionalizing the PDA surface with an aptamer that specifically binds only to *Salmonella*^7^. Specifically, a *Salmonella*-specific DNA aptamer was chemically conjugated to the PDA liposome via carbodiimide 1-ethyl-3-(3-dimethylaminopropyl)carbodiimide in combination with N-hydroxysuccinimide (NHS-EDC) reactive intermediate (carbodiimide method) before the UV-assisted polymerization process, as illustrated in Fig. 6a. Figure 6a also represents the mechanism of the colorimetric sensing of PDA liposome-aptamer sensor for *Salmonella typhimurium* detection. Initially, the PDA liposome-aptamer solution exhibits a blue color. When the bacteria of S. *typhimurium* are introduced, the color changes to red due to the strong binding of the aptamer to the bacteria, which induces distortion of the π-conjugation in the PDA backbone, thereby affecting the increase in the energy gap structure. Various essential parameters were also investigated, including aptamer concentration, reaction time, analyte concentration dependence, detection limit, recovery, and specificity. The results showed that the aptamer-functionalized PDA liposome in a solution system exhibits outstanding sensing performance.

Additionally, Wen et al. developed a PDA colorimetric sensor strip by functionalizing a PDA-coated polyvinylidene fluoride (PVDF) membrane with a DNA aptamer to detect Zn^2+^ ions in aqueous solutions^139^. A carbodiimide method was employed to conjugate the Zn^2+^-specific aptamer to the PDA monomer before the polymerization reaction. Moreover, their work revealed that the aptamer and linker lengths, as well as the aptamer structures, affect the colorimetric sensing performance. Interestingly, a more extended aptamer sequence and the hairpin aptamer structure exhibited outstanding colorimetric performance for Zn^2+^ detection. This may correspond to increased steric hindrance, leading to more sensitive color change when the aptamer length increases and the structural conformation changes from a closed loop to an open loop hairpin upon Zn^2+^ binding. Recently, Zhong and colleagues engineered a magnetic beads-capture aptamer-bacteria-detection aptamer-PDA sandwich structure for detecting foodborne pathogens, including *E. coli* O157:H7, *S. Typhimurium*, and *V. parahaemolyticus* in an aqueous solution system^43^. Specifically, the magnetic beads-aptamer is beneficial for bacterial enrichment before detection, thereby improving sensitivity. The functionalization strategy of an aptamer to PDA involves covalently binding the aptamer to magnetic beads and aptamer-DNA conjugation through the carbodiimide method. Specifically, optimization conditions include capture aptamer density, magnetic bead concentration, incubation time, and the detection aptamer ratio. This PDA sensor showed high selectivity only toward the target bacteria. Zhou and co-workers had also developed a similar approach by modifying the PDA coating on PVDF paper strips with an aptamer via carbodiimide reaction to detect *Bacillus thuringiensis* HD-73 spores^44^. The incubation time and specificity tests were also examined. Furthermore, a bidentate aptamer functionalized-PDA liposome sensor was fabricated to detect the thrombin protein^140^. The bidentate approach significantly improved the sensitivity and specificity due to the aptamer’s two distinct exosite recognition capabilities compared to the single aptamer. Instead of using an aptamer, the probe oligonucleotide was functionalized to a PDA liposome for genetic disease detection^141^. Specifically, the probe oligonucleotide is attached to the PDA backbone through hydrophobic attraction between the chains. This work demonstrated that an optimized molar ratio between the monomer reagents and the probe DNA is crucial for obtaining highly distinct color changes. Besides, antibodies were utilized to enhance the colorimetric performance of PDA sensors. Kim and Lee fabricated PDA-based liposomal biosensors by functionalizing them with the CD68 antibody to improve the sensitivity and selectivity for detecting exosomes^142^. Using the carbodiimide method, an anti-CD 63 monoclonal antibody was conjugated to the pre-hybridized PDA monomer.

On the other hand, small amino acid molecules or long peptide sequences were also employed to modify the PDA sensors. Yang and colleagues utilized histidine functionalized-PDA to detect Pb^2+^ ions or volatile organic compounds (VOCs) colorimetrically^69^. The imidazole unit in histidine could effectively serve as a strong ligand for metal ion recognition, thereby disturbing the PDA backbond and changing the color. Meanwhile, high exposure of VOCs to the histidine-PDA sensor could also promote the deformation of the PDA backbone, leading to a colorimetric response. The histidine-PDA sensor was prepared by reacting histidine-functionalized diacetylene (his-DA) with PCDA at an optimized molar ratio of 4:1. The results showed that the histidine-PDA sensor exhibited superior selectivity toward Pb^2+^ but poor selectivity toward various volatile organic compounds (VOCs). Meanwhile, a peptide was employed as the receptor or molecular recognition element in the PDA sensor for the selective and sensitive detection of trinitrotoluene (TNT)^39^. They also investigated multiple parameters influencing the chromatic response, including the end-group motif size, receptor surface density, and alkaline side chain length. This work revealed that the tripeptide (tripsin-histidine-tripsin) could act as a minimal binding moiety for recognizing TNT through a multivalent binding mode, as well as effectively perturbing the conjugated π-bond in the PDA backbone. Hence, to obtain a superior peptide-functionalized PDA platform, PDA system parameters (receptor and alkene length), composition (surface density control), and polymerization (assembly) process should be highly considered. Moreover, Kumar and his colleagues developed a tripeptide-based Zn(II) complex receptor unit functionalized with PDA as the sensing probe to detect adenosine triphosphate (ATP) in aqueous solution^143^. Specifically, the receptor unit, consisting of a complex tripeptide (phenylalanine-phenylalanine-glutamic acid-Zn), was covalently bonded with a polymer backbone via a peptide linkage using carbodiimide reaction. The preliminary assessment revealed that the developed PDA liposome exhibited distinct color changes, indicating high specificity towards ATP compared to other anions.

Besides, lipid derivatives were employed to functionalize PDA for a specific sensor. Ma et al. utilized glycolipids as receptor molecules conjugated to the PDA sensor through a physical approach to detect *E. Coli*^144^. Meanwhile, Weston et al. utilized phospholipid to functionalize PDA for detecting the free fatty acid (FFA) content in almond milk products^145^. The sensor detects the presence of FFA through a hydrophobic interaction between the alkyl tail of FFA and a moiety of the PDA membrane. Interestingly, the phospholipid functionalization of PDA significantly enhanced the chromatic response. Recently, 5-hydroxy-N^1^, N^3^-bis(pyridin-2-ylmethyl)isophthalamide (HP), a ligand molecule, was functionalized to PDA monomer, followed by a UV-assisted polymerization process to obtain a highly selective PDA sensor for cadmium ion (Cd^2+^) detection^146^. The sensing mechanism is based on the high affinity of the Schiff base of PDA-HP for Cd^2+^ to form a cadmium-complexed chelation process. Moreover, Davis and his colleagues fabricated PDA-embedded electrospun nanofibers functionalized with biotin biorecognition receptors for detecting protein^104^. Embedding PDA in a nanofiber platform could enhance the specific surface area, thereby improving the sensitivity. The result showed a strong interaction between biotin and streptavidin, inducing the chromatic change and enabling fast and selective colorimetric protein detection. Table 2 presents the functionalization strategies of PDA colorimetric sensors using various ligands or specific (bio)receptors, along with their corresponding analytical performances.

**Table 2:** Functionalization strategies with various ligands or specific (bio) receptors in polydiacetylene (PDA)-based colorimetric sensors and their analytical performances

Target analyte	Specific (bio)receptors	Detection technique	Sensing time (min)	Linear range	Limit of detection	References
*Salmonella Typhimurium* bacterium	Aptamer	Colorimetric (CR% value)	15	10^3^–10^7^ CFU mL^−1^	10^3^ CFU mL^−1^	^7^
Zn^2+^	Aptamer	Colorimetric (RCS% value)	240	0–1000 µM	125 µM	^139^
*E. coli* O157:H7, *S. Typhimurium*, *V. parahaemolyticus*	Aptamer	Colorimetric (CR% value)	30	*E. coli* O157:H7 (10–10^7^ CFU mL^−1^), *S. Typhimurium* (10─10^6^ PFU mL^−1^), *V. parahaemolyticus* (10–10^6^ PFU mL^−1^)	*E. coli* O157:H7 (39 CFU mL^−1^), *S. Typhimurium* (60 PFU mL^−1^), *V. parahaemolyticus* (60 PFU mL^−1^)	^43^
*Bacillus thuringiensis* HD-73 Spores	Aptamer	Colorimetric (CR% value)	240	3 × 10^7^–3 × 10^11^ PFU mL^−1^	3 × 10^7^ CFU mL^−1^	^44^
Thrombin	Aptamer	Colorimetric (CR% value)	15	0–10 µM	0.5 µM	^140^
Genes	Oligonucleotide	Colorimetric (CR% value)	NA	0–20 µM	NA	^141^
Exosome	Antibody	Colorimetric (CR% value) and fluorescence	30	3 × 10^7^–1 × 10^10^ vesicles mL^-1^	3 × 10^8^ vesicles mL^−1^	^142^
Pb^2+^ or volatile organic compounds (VOCs)	Amino acids (histidine)	Colorimetric (CR% value) and fluorescence	0.12	0–37 μM	Pb^2+^ (0.04 µM)	^69^
Trinitrotoluene (TNT)	Peptide	Colorimetric (CR% value)	NA	5.2–33.6 µM	NA	^39^
Adenosine triphosphate (ATP)	Tripeptide-based Zn (II) complex	Colorimetric (absorbance)	NA	NA	NA	^143^
*E. Coli*	Glycolipids	Colorimetric (absorbance)	NA	NA	NA	^144^
Free fatty acid (FFA)	Phospholipid	Colorimetric (CR% value)	1440	0–2.5 mg mL^−1^	NA	^145^
Cd^2+^	Ligand chelator	Colorimetric (absorbance) and fluorescence	1	NA	NA	^146^
Streptavidin (protein)	Biotin	Colorimetric (absorbance) and fluorescence	1	NA	NA	^104^

Colorimetric response percentage (CR%), red chromatic shift percentage (RCS%), absorbance, and fluorescence are typically used as the detection methods or parameters.

In summary, functionalization with specific ligands or bioreceptors, particularly aptamers and antibodies, offers a major advantage due to their high binding affinity and specificity, which help to minimize cross-reactivity in complex matrices such as food or clinical samples^137,138^. However, this strategy also presents notable drawbacks, including the high cost and potential instability of biological receptors (e.g., antibody or enzyme denaturation caused by temperature, pH fluctuations, or UV exposure)^147–150^. The functionalization process itself (e.g., carbodiimide coupling via EDC/NHS) must be carefully optimized to achieve consistent and efficient modification. Despite these limitations, this approach holds strong potential as a highly selective, sensitive, and user-friendly colorimetric sensing platform, particularly for biomedical applications.

### Nanomaterials

An equally promising surface functionalization strategy involves the integration with nanomaterials^127,151^. This is related to the distinctive characteristic of the used materials. Nanomaterials have an extensive specific surface area, allowing them to interact with analytes over a larger surface area^152–154^. In addition, nanomaterials are also easily modified to adjust their physicochemical properties, allowing for high customization to interact with specific analytes^155,156^. The combination of a large specific surface area of nanomaterials and the ease of modifying their properties enables them to interact well with target molecules^157–159^, thereby improving the performance of the fabricated PDA colorimetric sensors^160–162^.

Among the various nanomaterials employed to enhance colorimetric sensor performance, nanoparticles (NPs) are the most widely utilized due to their ease of fabrication and integration^163,164^. Nanoparticles with specific capabilities, such as gold nanoparticles (AuNPs), silver nanoparticles (AgNPs), and quantum dots (QDs), have been widely utilized in recent years to enhance the performance of colorimetric sensors^163,165–168^, including PDA-based colorimetric sensors. The combination of the sensor base’s colorimetric response and the functional capabilities of AuNPs, AgNPs, and QDs can produce colorimetric sensors with excellent performance for various applications, including the detection of specific biomolecules and diseases, hazardous pollutants, VOC gases, and food freshness and quality.

AuNPs are the most widely used to improve the performance of colorimetric sensors. Most uses of AuNPs utilize the local surface plasmon resonance (LSPR) property that can enhance the response signal in calorimetric detection^169,170^. Additionally, the surface of AuNPs can be easily modified with aptamers^171^, antibodies, enzymes, proteins^172^, and DNA^173^, enabling them to interact more effectively with target analytes. In its colorimetric applications, AuNPs are widely used to detect various substances, including heavy metals^47,174–176^, biological molecules^177,178^, and pharmaceutical compounds^179,180^.

**Fig. 7:**
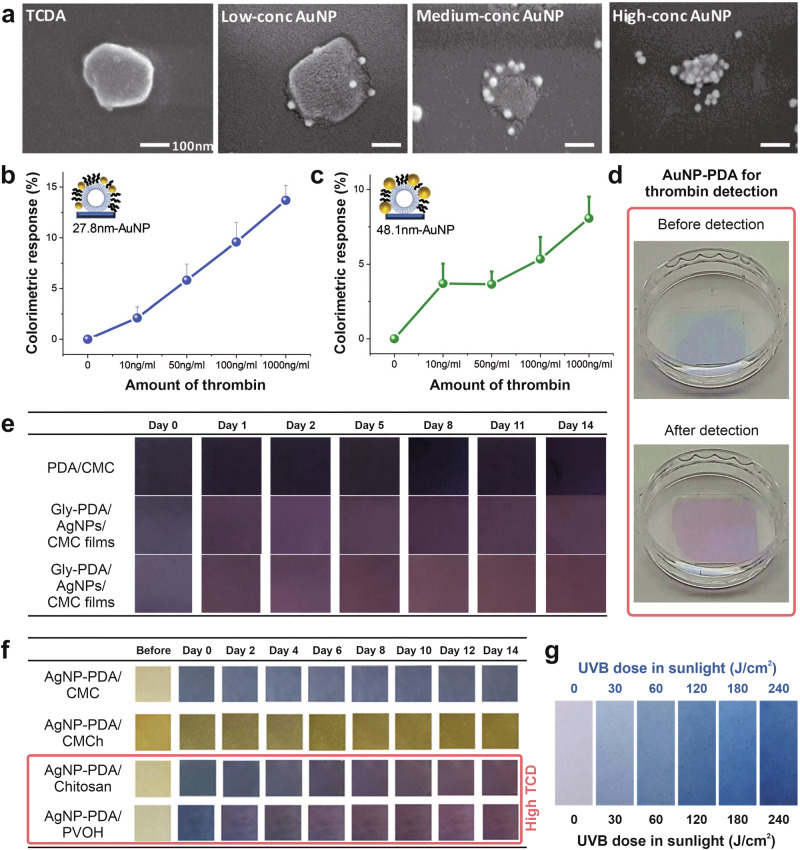
Surface functionalization of polydiacetylene (PDA)-based colorimetric sensors using nanomaterials. **a** SEM image of thrombin-binding-aptamer (TBA)-AuNP-PDA liposomes with various amounts of AuNP concentration of 6.6 μM (low), 20 μM (medium), and 26.5 μM (high). As the concentration of AuNPs increased, the liposome structure appeared to shrink and deform, likely due to mechanical stress induced by AuNP particles bound to the surface of the PDA liposomes. **b**, **c** Colorimetric responses of grafted 27.8 nm and 48.1 nm AuNPs, respectively, illustrate the relationship between AuNP size/concentration and the rate of color shift. **d** The colorimetric reactions of the AuNP–PDA sensor toward thrombin show a blue-to-red transition that is visible to the naked eye. Reproduced with permission from ref. ^40^, copyright (Royal Society of Chemistry, 2022). **e** Comparative color changes of PDA, PDA/AgNP, and glycerol (Gly)-PDA/AgNP-embedded carboxymethyl cellulose (CMC) films over 14 days demonstrate an enhanced thermochromic sensitivity induced by the AgNP incorporation. Reproduced with permission from ref. ^187^, copyright (Multidisciplinary Digital Publishing Institute, 2020). **f** Observations of the colorimetric response of AgNP–PDA in various biodegradable polymer films show distinct chromatic and thermal sensitivities attributed to AgNP incorporation. This enhancement is influenced by molecular interactions between the carboxylate and hydroxyl groups of PDA and AgNPs, which induce structural rearrangement through van der Waals forces and hydrogen bonding. Adapted with permission from ref. ^188^, copyright (Elsevier B.V., 2024) **g** Photographs of a colorimetric PVA-based film using DA(8,12)/Zn²⁺/ZnO-2.2 nm nanocomposites depict the average absorbance at 640 nm under varying sunlight irradiation times (UV-B doses). The quantum confinement effect of ZnO QDs significantly enhances sensitivity to UV-B irradiation. Adapted with permission from ref. ^193^, copyright (Elsevier B.V., 2024).

Due to their high flexibility, AuNPs can also be integrated with PDA to enhance colorimetric performance. For example, Kim et al. used AuNPs on PDA liposomes as molecular binding signal sensitizers, providing additional conformational distortion to the PDA backbone structure through steric repulsion with the attached biomolecules^40^. In their study, AuNPs and thrombin-binding aptamers were functionalized on PDA liposomes, which were attached to a substrate to detect thrombin. The concentration of AuNPs was varied from low to high to assess its effect on colorimetric performance (see Fig. 7a). In this system, the presence of AuNPs not only enhances the stability of the PDA structure but also amplifies the extent of the color change. As a result, the use of AuNPs with a diameter of 27.8 nm at a medium concentration yields a higher response than those with a diameter of 48.1 nm, as depicted in Fig. 7b, c, and can increase the sensor’s sensitivity by up to 2.5 times compared to the sensor without AuNPs. The addition of AuNPs causes increased morphological pressure on the backbone structure, which then disrupts and destroys the structural planarity, allowing for a more significant colorimetric response^33,181^. Visually, the color change of the colorimetric sensor can be clearly observed with the naked eye, transitioning from blue to red, as illustrated in Fig. 7d.

In addition to AuNPs, the use of AgNPs as a colorimetric sensor exhibits significant potential due to their antimicrobial properties and surface energy^182^. Similar to AuNPs, the performance of AgNPs-based colorimetric sensors is also exciting. Several studies have demonstrated that AgNPs can serve as colorimetric sensors to detect metal ions, such as Pb^2+^ and Hg^2+^, with promising performance^183,184^. In addition, AgNP-based colorimetrics are also widely applied in pharmaceuticals, biomolecules, and the detection of environmental contaminants^168,185,186^.

Functionalization of the PDA matrix using AgNPs also demonstrates the potential and promising performance of colorimetric sensors for use as time-temperature indicators. For example, Saenjaiban et al. conducted a study to investigate the effect of AgNPs and glycerol on the color change of PDA/AgNPs embedded in CMC film in its application as a time-temperature indicator^187^. The observation showed that the CMC film made with a PDA/AgNP ratio of 1:3 (v/v) had a faster discoloration time compared to other variations. At 35 °C, the PDA/AgNP film exhibited a color change from purplish blue to purple and then to reddish-purple over time, as presented in Fig. 7e. This response to temperature is attributed to the addition of AgNPs. AgNPs are good thermal conductors that can help PDA withstand temperature changes. In addition, AgNPs can increase the surface area of PDA. Hence, a larger area can be exposed to the temperature. The combination of these two factors makes the PDA/AgNPs film more sensitive to temperature fluctuations, resulting in a faster response. This finding is beneficial for applications in the fruit and vegetable industry, especially for quality monitoring and control.

Senjaiban et al. also conducted another study related to time-temperature indicators using AgNPs^188^. This study aimed to produce biodegradable polymer films based on carboxymethyl cellulose, polyvinyl alcohol (PVOH), chitosan, and carboxymethyl chitosan (CMCh), integrated with 10% (w/w) AgNP-PDA, to investigate their thermochromic properties. The observation at 35 °C for 14 days revealed a color change from blue to reddish purple and reddish brown for AgNP-PDA on chitosan and PVOH-based films, with the highest sensitivity values, respectively, as shown in Fig. 7f. This study can be utilized for temperature tracking of fresh products at low temperatures.

The other nanoparticles that have attracted considerable attention and hold great potential for application in colorimetric sensors are quantum dots (QDs). QDs are semiconductor nanoparticles with a very small size, typically ranging from 2 to 10 nm^189^. QDs have unique optical properties due to the quantum confinement effect^190^. The utilization of QDs is based on their ability to emit bright and stable fluorescence in various environments^190^. Therefore, QDs are considered ideal for optical transduction in sensors. In addition, QDs can also provide visual output in the form of color changes when exposed to specific analytes or other stimuli such as pH and temperature^190^. This can occur due to changes in the surface structure of the QDs, which affect their absorption spectrum and results in a color shift that can be directly detected. Another advantage of QDs is their ease of fabrication and low cost. QDs are also easily surface-modified by functionalizing with various ligands or receptors, allowing them to be applied as sensors that are selective for specific analytes^191,192^. As a colorimetric sensor, QDs have also demonstrated superior performance. Several studies have shown their colorimetric performance in detecting Cu(II), Mn(II), Hg(II), Pb(II), As(III), and Cd(II).

The use of QDs can also be combined with PDA, such as the research conducted by Siriboon et al., to detect UV-B light^193^. Their study investigated the effect of ZnO QD size on the sensitivity of their response to UVB. That research revealed that the size of ZnO QDs controls sensitivity. The incorporation of ZnO QDs with a size of 2.2–3.8 nm showed the highest sensitivity to UV-B light, as depicted in Fig. 7g. Not only the influence of ZnO QDs but also the variation of the alkyl chain length of the DA monomer can be utilized to adjust the sensor’s sensitivity to ultraviolet B (UVB). The observed phenomenon can occur due to two factors experienced by ZnO QDs and DA. The quantum confinement effect inherent in ZnO QDs governs its absorption region, while variations in alkyl chain length affect the color transition behavior of the nanocomposite when exposed to UVB radiation.

The integration of nanomaterials is a highly effective strategy for enhancing signal amplification and improving sensor stability. Their high specific surface area increases the available interaction sites for analytes, thereby improving the efficiency of target recognition^157–159^. Nanomaterials such as AuNPs can significantly enhance sensitivity through mechanisms like LSPR and energy transfer^169,170^, while others, including ZnO and AgNPs, contribute to improved thermal stability and faster response times^187,193^. The primary advantage of this strategy is the substantial increase in signal intensity. Despite its promise, this approach also presents several limitations, including fabrication complexity, the risk of nanomaterial aggregation, and higher overall costs^40,168,194,195^. Environmental-impact and biocompatibility concerns associated with certain nanomaterials must also be carefully evaluated. This strategy is particularly suitable for applications requiring extremely low detection limits or enhanced physical stability, such as environmental monitoring, food safety, and medical diagnostics. Moreover, its potential can be further expanded through the development of nanohybrid sensors capable of simultaneously detecting multiple analytes. The growing integration of electronic systems and Internet of Things (IoT) technologies also support the incorporation of PDA-based nanomaterials into real-time, practical, and portable sensing platforms, making them increasingly responsive to industrial and market needs.

### Others

**Fig. 8:**
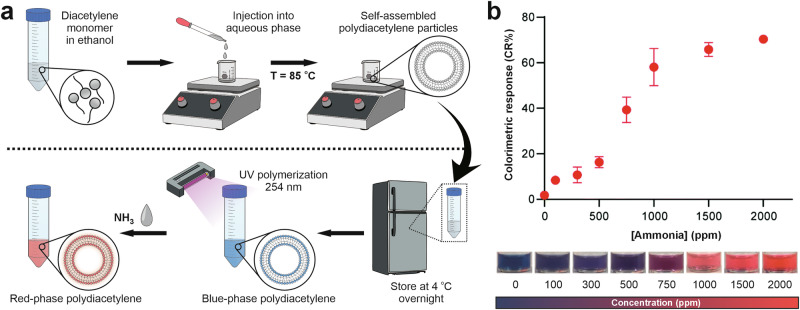
PDA-based colorimetric sensors for ammonia detection. **a** Schematic illustration of self-assembled PDA vesicles prepared using the self-injection method. The DA monomer was first dissolved in ethanol and then injected into an aqueous phase at 85 °C to form self-assembled polydiacetylene particles. After cooling overnight, polymerization was initiated under UV irradiation (254 nm) to obtain blue-phase PDA. Upon exposure to ammonia vapor (NH₃), the PDA structure underwent a conformational change, producing a color shift from blue to red due to disruption of the π-conjugated backbone. **b** The colorimetric response of PDA to various ammonia concentrations (0–2000 ppm) shows a progressive color change from blue to red with increasing concentration, confirming the high sensitivity of PDA vesicles as a colorimetric sensing platform. Adapted from ref. ^201^, copyright (Royal Society of Chemistry, 2020).

Apart from the primary functionalization strategies discussed above (i.e., using chemical groups, ligands, bioreceptors, and nanomaterials), several other methods have been investigated to improve the performance of PDA-based colorimetric sensors. One notable approach involves the formulation of PDA in the form of lipid vesicles (see Fig. 8), particularly in the development of colorimetric sensors for biological sample monitoring. This strategy is favorable due to the ability of PDA to mimic the structural properties of biological membranes, thereby facilitating effective interaction with bacteria and viruses. Furthermore, PDA provides a versatile platform for functionalization with biomolecular recognition elements, such as peptides, nucleic acids, or antibodies, allowing selective detection in biological environments^64^. Several studies have reported the synthesis of PDA vesicles using self-assembly and UV polymerization methods, typically starting from DA monomers, such as PCDA. These monomers will interact with lipid molecules (e.g., dipalmitoylphosphatidylcholine (DPPC) and dimyristoylphosphatidylcholine (DMPC) through self-assembly in an aqueous solution. After the formation of multilamellar vesicles, the mixture is irradiated with ultraviolet light (UV, wavelength around 254 nm) to initiate the polymerization reaction of diacetylene, resulting in a dark blue PDA. Although self-assembly enables the spontaneous formation of vesicles, the size and distribution of these vesicles are often polydisperse, affecting the consistency of the color signal. Kang et al. studied the synthesis of PDA with various types of phospholipids, namely 1,2-dimyristoyl-sn-glycero-3-phosphocholine (DMPC), 1,2-dimyristoyl-sn-glycero-3-phosphate (DMPA), and 1,2-dimyristoyl-3-trimethylammonium-propane (DMTAP), which have different head charges and phase transition temperatures (Tm) in self-assembly^196^. This study demonstrates that combining PCDA-epoxy as a monomer with charged phospholipids enables control over the PDA surface charge and the size formed. DMPA (anionic phospholipid) has a significant role in the characteristics of the zeta charge, charge control, and flexibility of the formed PDA membrane. This phenomenon occurs because DMPA can prevent aggregation due to the presence of high electrostatic repulsion forces, resulting in the production of particles with a smallest size of approximately 187 nm.

In contrast to neutrally charged DMPA, the use of this uncharged phospholipid causes rapid and unstable aggregation. The vesicles were then conjugated with antigens from bovine viral diarrhea virus (BVDV), followed by a blocking process using bovine serum albumin (BSA). After UV polymerization, the system was tested to detect BVDV antibodies at concentrations ranging from 0.001 to 100 μg mL^−1^. The sensor response was determined through the colorimetric response (CR) parameter, calculated by comparing the absorbance intensities of the blue (~640 nm) and red (~540 nm) bands before and after antibody interaction. The test results showed that the combination of DMPA gave the best performance. The weak lipid packing in DMPA causes the membrane structure to be more flexible, making it easy to experience π-conjugation distortion when the antibody binds to the antigen on the vesicle surface. However, another disadvantage of PDA vesicles is that they tend to experience color fading or degradation under extreme environmental conditions such as low pH, high temperature, or prolonged light exposure. This can reduce sensitivity and accuracy of detection^197–199^. Therefore, to improve this stability, PDA vesicles with covalent connections to form 3D structures have been developed and studied^92^. This structure provides higher mechanical and chemical stability compared to ordinary supramolecular self-assembly, making it suitable for biosensor applications in complex environments^200^.

Alternatively, PDA vesicle synthesis can also be carried out via a solvent injection method. Tang et al. reported that this method represents a novel approach distinct from conventional techniques, such as thin-film hydration^201^. The solvent injection method facilitates vesicle formation by rapidly mixing organic and aqueous phases, thereby enhancing stability. An illustration of this solvent injection method to create PDA vesicles is shown more clearly in Fig. 8a, b. In this procedure, diacetylene (DA) monomer is first dissolved in a polar solvent, such as ethanol. The resulting solution is then slowly injected into an aqueous medium at a temperature above the monomer-phase transition point, while being stirred rapidly. After the injection process is complete, the mixture is incubated to allow ethanol evaporation, enabling the amphiphilic monomer to form a vesicle structure spontaneously. The formed vesicles are then cooled and photopolymerized using 254 nm UV light to produce a blue-phase PDA that is sensitive to stimuli, such as ammonia gas (see Fig. 8a). The results of the study demonstrated that the PDA sensor exhibited a visible color change at ammonia concentrations above 500 ppm within 60 min (see Fig. 8b). Additionally, the solvent injection method was successfully employed for large-scale PDA synthesis (up to 240 mL), yielding consistent particle size and good long-term stability. After being stored for four months, there was no significant change in the particle size distribution or zeta potential value. The result indicates that the vesicle structure remains stable over a prolonged period, which is a considerable advantage for practical applications^201^. During this injection phase, the simultaneous interaction between the organic solvent and water favors the thermodynamic formation of vesicles. This efficient self-organizing process does not require secondary procedures such as sonication or extrusion. The advantages of this method were confirmed by Tjandra et al., who showed that the self-assembly process was successful^76^. Conditions such as solvent type, injection rate, and syringe diameter affected the particle size, morphology, and color intensity of the PDA without altering its thermochromic sensitivity or pH. However, control over the solvent injection method still requires careful optimization of its parameters. For example, Tjandra et al. also reported that solvents that are not miscible with water, such as chloroform, suppress vesicle formation due to phase instability^76^. Additionally, high injection rates tend to produce PDA particles with higher color intensity, as they facilitate the formation of dense topochemical polymerization regions. This highlights the importance of a deeper understanding of the phase dynamics and kinetics during injection, which directly impact the quality of the final sensor^76^.

In summary, the main advantage of this approach lies in its ability to adjust the intrinsic properties of PDA sensors without introducing external chemical or biological components, thereby simplifying the overall synthesis process^12,33,197^. However, PDA in vesicle form is susceptible to degradation under extreme conditions (e.g., very high or low pH), as well as challenges in achieving high uniformity on a large scale^197–199^. Thus, this strategy not only offers simplicity in the synthesis process but also serves as an alternative approach that opens new opportunities for developing reliable sensors.

## Performance evaluation and current challenges

The development and application of PDA-based colorimetric sensors have advanced considerably. Nevertheless, several challenges remain that hinder their broader practical applications. These include quantitative analysis limitations, the fabrication and functionalization complexity, scalability, and cost-efficiency. Lighting factors often influence quantitative analysis limitations, such as differences in digital colors that smartphones produce. This indicates the need for calibration and standardization protocols for digital color analysis. Scalability is also a significant concern. Although inkjet printing offers ease and flexibility in patterns^28,29^, reproducing uniform sensors with consistent functionalization on a large scale is not easy. Control is needed for each monomer purity variation and the functionalization process’s success between batches.

Additionally, cost efficiency must be carefully considered. For example, the use of functionalization agents involving specific receptors (e.g., aptamers, antibodies)^202^ or nanomaterials (e.g., AuNPs)^40,194^, improves sensor performance but also can increase production costs, thereby limiting its use in resource-constrained environments. Specific receptors demonstrate excellent analytical performance for various targets (see Table 3), but often involve the most complex and expensive functionalization routes. For example, the use of PDA liposomes conjugated with aptamers for pathogen detection provides high selectivity^43^, but the synthesis process is complex and costly. Similarly, sensors modified with AuNPs exhibit higher sensitivity^40,194^, but the material cost is much higher, and the fabrication complexity far exceeds that of chemically functionalized sensors. Therefore, careful control over the functionalization process, suitable material selection, and optimized fabrication techniques are necessary to achieve high-performance PDA-based sensors. The different functionalization strategies for PDA-based colorimetric sensors are compiled in Table 3, which also highlights the corresponding PDA monomers, target analytes, fabrication techniques, and sensor performance outcomes.

**Table 3:** Comparison of various functionalization strategies for PDA-based colorimetric sensors

Functionalization strategy	Fabrication method	Type of used PDA	Functionalization agent	Target analysis	Reaction time	Linear detection range	LOD	Refs.
Chemical functional group	Drop casting	PCDA	Imidazole	Fe^3+^	10 min	3 × 10^7^–10^11^ CFU mL^−1^	9 μM	^13^
Chemical functional group	Drop casting	PCDA	Imidazole	NADPH	3 min	7–245 μM	23.5 nM	^72^
Chemical functional group	Wax screen printing	PCDA	Amine-terminated linker	dsDNA	1 min	0–1.8 μM	10 nM	^202^
Chemical functional group	Sonnication self-assembly	PCDA	Phenylboronic acid	Pb^2+^	10 s	10–10 nM	0.1 μM	^133^
Chemical functional group	Self-assembly	PCDA	9-Aminoacridine (9AA)	dsDNA	60 min	0–30 μM	20 nM	^86^
Chemical functional group	Self-assembly	PCDA	Diaminotriazine	Thymine ^,^Oligothymidine	60 min	1–100 nM	12.6 nM^,^14.6 nM	^135^
Chemical functional group	Sonnication self-assembly	PCDA	4-Hydroxybenzaldehyde and malonitrile	CN^-^	5 min	0–1500 μM	0.55 μM	^27^
Chemical functional group	Sonnication self-assembly	PCDA	Galloyl group	Pb^2+^	5 min	0–10 μM	1.329 μM	^82^
Chemical functional group	Self-assembly	PCDA	Alendronate	Pb^2+^	1 min	0–10 μm	83 μm	^203^
Chemical functional group	Sonnication self-assembly	PCDA	Sodium alendronate	Biogenic polyamines: ^,^Spermine (SP)^,^Spermidine (SPD)^,^Putrescine (PUT)^,^Cadaverine (CAD)	1 min	0.1–1 μM (SP & SPD)^,^1 – 10 Μm (PUT & CAT)	4.33 ppb (SP)^,^5.1 ppb (SPD)^,^10.1 ppb (PUT)^,^10.7 ppb (CAD)	^88^
Ligand	Carboiimide method	PCDA	Aptamer	*E coli* O157:H7 *S.typhimurium*^,^*V.parahaemolyticus*	30 min	10–10^7^ CFU mL^−1^^,^10–10^6^ CFU mL^−1^^,^10–10^6^ CFU mL^−1^	39 CFU mL^−1^^,^60 CFU mL^−1^^,^60 CFU mL^−1^	^43^
Ligand	Impregnation	PCDA	Fibrinogen	Staphylococcus aureus	60 min	0–6.5 × 10^6 ^CFU mL^−1^	65 CFU mL^−1^ (in pure culture)^,^50.1 CFU mL^−1^ (in food matrices)	^204^
Ligand	Self-assembly	PCDA	GSH tripeptide	GST-fusion protein	15 min	0–50 μM	13.4 μM	^205^
Ligand	Drop casting	PCDA	Histidine	Pb^2+^	7 s	0–37 μM	0.04 μM	^69^
Ligand	Self-assembly	PCDA	cDNA-urease	Microcystin-LR	60 min	5–100 ng mL^−1^	1 ng mL^−1^	^206^
Ligand	Self-assembly	PCDA	Anti-CD63 monoclonal antibody	Exosome	30 min	1 × 10^8^–3 × 10^9^ vesicles mL^−1^	3 × 10^8^ vesicles mL^−1^	^142^
Ligand	Drop casting	PCDA	Streptavidin-biotin-modified DES	Diethylstilbestrol (DES)	60 min	2–1250 ng mL^−1^	0.85 ng mL^−1^ (smartphone-based)^,^10 ng mL^−1^ (naked-eye, paper-based sensor)	^207^
Ligand	Thin film hydration	TCDA	Anti-AFB1	Aflatoxin B1 (AFB1)	5 min	5–50 ppb	17.3 ppb	^208^
Ligand	Self-assembly	TCDA-NHS	Aptamer	Salmonella typhimurium	15 min	5–50 ppb	1 × 10^3^ CFU mL^−1^	^7^
Ligand	Self-assembly	TCDA-NHS	Aptamer	Malignant exosomes	15 min	1 × 10^3^ – 10^7^ CFU mL^−1^	3.66 × 10^4^ particles μL^-1^	^83^
Ligand	Solvent evaporation method	TCDA-NHS	Aptamer	Bacillus thuringiensis spores	60 min	7 × 10^4^–10^5^ particles mL^−1^	3 × 10^7^ CFU mL^−1^	^44^
Nanomaterials	Sonnication self-assembly	PCDA	Zn^2+^/ZnO	CTAB	1 min	0–50 mM	0.5 mM	^41^
Nanomaterials	Drop casting	PCDA	ZnO	Propionic acid	30 s	0.1–667 mM	10 mM	^90^
Nanomaterials	Microarray spotting	PCDA	AuNPs	Human immunoglobulin E	30 min	0.01–10.000 ng mL^−1^	0.1 ng mL^−1^	^194^
Others	Spray coating	PCDA	Iron chloride	H_2_O_2_	240 min	3–94 ppm	0.2–3 ppm	^1^

Main functionalization strategies involve chemical functional groups, ligands, and nanomaterials.

## Conclusions and future perspectives

Due to innovative functionalization strategies that greatly enhance sensitivity, selectivity, and stability, PDA-based colorimetric sensors have emerged as a versatile and compelling technology. Key developments include the use of specific ligands or receptors (e.g., aptamers for pathogen detection), chemical modifications (e.g., polyamines for CO₂ detection, thiol groups for formaldehyde sensing), the incorporation of nanomaterials (e.g., AuNPs and ZnO QDs for signal amplification), and other approaches (such as the formation of PDA lipid vesicles) as alternative innovations. This review highlights that each strategy has distinct characteristics and advantages that can be optimized based on the respective target application. Chemical modifications offer an efficient and straightforward means of detecting analytes through specific interactions (e.g., hydrogen bonding), specific ligands or receptors provide high affinity toward target biomolecules, the integration of nanomaterials enhances both optical response and system stability, and vesicle formation contributes simplicity of design and flexibility in sensor architecture.

Currently, PDA-based colorimetric sensors are used in a variety of fields, such as clinical diagnostics, food safety, and environmental monitoring. Even with significant progress, there are still issues with attaining high sensitivity and selectivity, guaranteeing stability over time, preserving quantitative accuracy, and facilitating economical production.

The selection of base materials has a direct impact on the effectiveness of these functionalization strategies. Because of their long alkyl chains and greater stability than their short-chain counterparts, monomers like PCDA and TCDA are widely used. In addition to the alkyl-chain length, the chemical properties of the DA monomer head group also play a crucial role in determining sensor performance. The head group acts as a recognition site capable of interacting specifically with target analytes through non-covalent interactions (e.g., hydrogen bonding, π–π stacking, van der Waals forces) or direct chemical reactions. Furthermore, choosing functionalization agents or supporting materials like ZnO or AuNPs is essential for boosting PDA structural integrity and improving overall sensor performance. Additionally, these components support enhanced long-term stability and adaptability in challenging environmental circumstances.

To ensure that active materials can be reliably deposited on sensors and meet application requirements, various fabrication techniques have been developed, such as drop casting, electrospinning, inkjet printing, and spin coating. While inkjet printing is appropriate for scalable production with flexible substrate options, spin coating allows for uniform film formation under controlled laboratory conditions. Moreover, electrospinning can create nanofibers with improved sensitivity for detection.

Future studies should focus on developing next-generation PDA materials and integrated technologies to overcome current limitations while enhancing environmental sustainability and performance. Innovations in functionalization can be pursued through hybrid approaches, such as combining specific chemical interactions with nanomaterial-based signal amplification or incorporating selective receptors into optimally designed vesicles to achieve superior selectivity and stability. In addition, the development of biodegradable variants and reversibly responsive PDA will enhance environmental compatibility and expand the range of potential sensor applications. Advances in fabrication technologies, including microfluidics and 3D printing, also offer promising opportunities to improve accuracy and scalability. Moreover, as demonstrated by recent smartphone-based platforms, integrating Artificial Intelligence (AI) and the Internet of Things (IoT) into PDA sensor systems can enable intelligent data processing and real-time monitoring. Finally, ensuring financial feasibility and environmental responsibility requires attention to sustainability and commercialization aspects. Progress in this field will depend on strong multidisciplinary collaboration among researchers, industry stakeholders, and policymakers. With continued development and integration, PDA-based sensors have the potential to evolve into reliable, portable, and cost-effective detection platforms capable of addressing global challenges in environmental monitoring, food safety, and healthcare.
